# Impact of Psychological Factors on Functional Performance among Patients with Chronic Obstructive Pulmonary Disease

**DOI:** 10.3390/ijerph20021285

**Published:** 2023-01-10

**Authors:** Monira I. Aldhahi, Baian A. Baattaiah, Rakan I. Nazer, Ali Albarrati

**Affiliations:** 1 Department of Rehabilitation Sciences, College of Health and Rehabilitation Sciences, Princess Nourah bint Abdulrahman University, P.O. Box 84428, Riyadh 11671, Saudi Arabia; 2 Department of Physical Therapy, Faculty of Medical Rehabilitation Sciences, King Abdulaziz University, Jeddah 21589, Saudi Arabia; 3 Cardiac Sciences Department, College of Medicine, King Saud University, Riyadh 11451, Saudi Arabia; 4 Rehabilitation Sciences Department, College of Applied Medical Sciences, King Saud University, Riyadh 11451, Saudi Arabia

**Keywords:** anxiety, depression, exercise intolerance, performance

## Abstract

The role of anxiety and depression in functional performance during walking in patients with chronic obstructive pulmonary disease (COPD) is controversial. In this cross-sectional study, we aimed to assess the effects of anxiety, depression, and health-related quality of life (HRQOL) on the functional performance of this patient population. Seventy COPD patients aged 63 ± 11 years participated in the study. To measure their functional performance, the six-minute walk test (6MWT) was used. Anxiety and depression were assessed using two questionnaires: the Anxiety Inventory for Respiratory Disease (AIR) scale and the Hospital Anxiety and Depression Scale (HADS). The St. George’s Respiratory Questionnaire (SGRQ) was used to assess HRQOL. Based on their anxiety levels, the patients were divided into a no anxiety group and a high anxiety group. There were no significant differences between the two groups in terms of pulmonary function profile or smoking status. The mean AIR and HADS (depression) scores were high (12.78 ± 4.07 and 9.90 ± 3.41, respectively). More than one-third of the patients (46%) reported high anxiety levels (above the standard cutoff score of 8). The mean score of the aggregated HADS scale was significantly higher in the high anxiety group (20.87 ± 6.13) than in the no anxiety group (9.26 ± 4.72; *p* = 0.01). Patients with high anxiety had poorer functional performance (6MWT: 308.75 ± 120.16 m) and HRQOL (SGRQ: 56.54 ± 22.36) than patients with no anxiety (6MWT: 373.76 ± 106.56 m; SGRQ: 42.90 ± 24.76; *p* < 0.01). The final multivariate model explained 33% of the variance in functional performance after controlling for COPD severity (*F* = 8.97). The results suggest that anxiety, depression, and poor health status are significantly associated with poor functional performance. This study highlights the need to screen patients with COPD at all stages for anxiety and depression.

## 1. Introduction

Chronic obstructive pulmonary disease (COPD) is a chronic lung disease with extrapulmonary manifestations [1]. Psychological disorders, including anxiety and depression, are among the most common comorbidities associated with COPD [2] and may have deleterious effects on patients’ functional performance and health-related quality of life [3,4]. Performing routine functional activities becomes increasingly challenging for patients with COPD as the severity of the disease increases. Patients with COPD report lower physical performance levels than healthy individuals and patients with other chronic diseases [5,6,7]. Reduced functional performance, in turn, alters patients’ lifestyles, leading to social isolation, anxiety, and depression [8,9]. Much progress has been made during the last two decades in understanding the management, and functional and psychological consequences of COPD. The psychological and physical health factors that impact the functional performance of patients with COPD are of great interest and warrant further investigation.

COPD patients have been shown to cover shorter distances than healthy individuals on six-minute walk tests (6MWT) [10]. Although several factors have been proposed to contribute to poor functional performance in COPD patients [11,12,13], the effects of psychological factors, such as anxiety and depression, on physical performance and the overall health status of COPD patients is controversial. Some studies have shown that anxiety and depression negatively impact physical functioning among patients with COPD [14,15], while another study found that the levels of anxiety and depression of COPD patients were not correlated with the distance walked during functional performance tests [16].

Reduced physical performance is known to cause a shift in patients’ lifestyles and to result in a vicious cycle of reduced exercise tolerance, which further reduces activity levels, leading to social isolation and depression [8,9]. Previous research has shown that anxiety and depression are among the serious comorbidities that affect the COPD population and can lead to negative impacts on patients’ quality of life [17,18,19,20]. Health status, or health-related quality of life, is another important factor that may impact the functional abilities of this population. Previous studies have found that a change in the overall health status of patients with COPD is concomitant with a change in exercise tolerance [21,22]. Several factors have been proposed to contribute to this decline in functional performance [11,12,23,24]. However, the contribution of anxiety, depression, and overall health status to the ability to walk requires further investigation.

Identifying the factors that affect functional performance may enable the development of effective interventions to improve the capacity of COPD patients to engage in physical activity and carry out daily life activities. Therefore, this study aimed to compare the functional performance and depression and health status of COPD patients with and without psychological anxiety. The study also aimed to examine the associations between psychological factors (anxiety and depression), health status, and walking performance. The results of this study may contribute to developing early psychological interventions and management strategies to minimize the effects of anxiety and depression on the functional performance of patients with COPD, thus improving their fitness levels and overall health.

## 2. Materials and Methods

### 2.1. Study Design and Settings

This cross-sectional study enrolled 70 clinically stable patients with COPD at King Saud University Medical City and King Fahad Medical City, Riyadh, Saudi Arabia, using non-probability convenience sampling. The study was approved by the respective institutional review boards (IRB_017E; 22-0522) and conducted according to the principles of the Declaration of Helsinki.

### 2.2. Participants

Patients with a confirmed diagnosis of COPD were eligible to participate in the study. The diagnosis was confirmed according to the Global Initiative for Chronic Obstructive Lung Disease (GOLD) criteria [25]. All prospective participants were informed about their rights and the purpose and procedures of the study and signed written informed consent forms. Participants with obesity, severe musculoskeletal disorders, cardiovascular diseases, non-COPD-related chronic inflammatory diseases, autoimmune diseases, other pulmonary diseases, psychotic disorders, and dementia or other neurological disorders were excluded from the study. Patients with a history of mental or physical disorders that interfered with their ability to understand and respond to questionnaires were also excluded.

### 2.3. Procedures

Screening for exercise appropriateness was performed according to the Physical Activity Readiness Questionnaire plus (PAR-Q^+^). Anthropometric measurements were performed using a standardized method. These included height in centimeters, weight in kilograms, measured using an electronic digital scale, and body mass index, calculated as weight divided by height squared. The assessments included spirometry, the Six-Minute Walk Test (6MWT), and three questionnaires.

### 2.4. Measures

#### 2.4.1. Pulmonary Function Test

Pulmonary function was assessed using spirmetry (Vitalograph Alpha 6000, Vitalograph Ltd., Buckingham, UK) according to American Thoracic Society (ATS) standards [26]. Post-bronchodilator values were used to confirm COPD. The variables obtained were forced vital capacity (FVC), forced expiratory volume in one second (FEV_1_), and FEV_1_/FVC ratio.

#### 2.4.2. Six-Minute Walk Test

The 6MWT was used to measure the patients’ functional performance. The 6MWT is valid and reliable and has been shown to correlate strongly and significantly with peak oxygen consumption, which is a proxy for cardiopulmonary exercise capacity (*r* = 0.73). Furthermore, it has been shown to be a more reliable indicator of a patient’s ability to perform daily activities than peak oxygen uptake [27].

Prior to testing, manual brachial blood pressure and heart rate were measured at rest. The patients were asked to walk in a 30-m-long enclosed flat corridor for six minutes. The test was conducted according to ATS guidelines [27]. The number of laps covered, distance, heart rate, and pulse oximetry were recorded simultaneously during the test.

#### 2.4.3. Psychological Status

Anxiety and depression were assessed using the Anxiety Inventory for Respiratory Disease (AIR) scale and the Hospital Anxiety and Depression Scale (HADS). The AIR scale comprises 10 items rated on a 4-point scale ranging from 0 (no symptoms of anxiety) to 3 (anxiety symptoms almost all of the time). A score summation of 8 or higher indicates severe anxiety symptoms. The psychometric properties of the AIR scale among Arabic-speaking patients with COPD have been validated previously [28,29,30]. The scale shows good psychometric properties of internal consistency coefficients (ICC(2,1) = 0.86; Cronbach’s α = 0.91) [30].

The HADS has been shown to have good reliability and validity in measuring anxiety and depression. It is a self-reported questionnaire comprising 14 items divided into two subscales: seven items for anxiety (HADS-A) and seven items for depression (HADS-D) [31]. Depression items include ‘“I have lost interest in my appearance:” and “I feel as if I am slowed down”; Anxiety items include “I still enjoy the things I used to enjoy”; and “Worrying thoughts go through my mind” The HADS has demonstrated good psychometric properties of internal consistency coefficients (Cronbach’s α for HADS-A = 0.83; for HADS-D = 0.77) [32]. The items are rated on a 7-point Likert scale. Scores higher than 8 and lower than 11 indicate probable depression or anxiety symptoms, and scores of >11 indicate clinically severe symptoms.

#### 2.4.4. Health-Related Quality of Life

The Arabic version of the St. George’s Respiratory Questionnaire (SGRQ) was used to assess health-related quality of life. The SGRQ is a self-reported questionnaire designed to measure the impact of COPD on overall health, daily life, and perceived well-being. It consists of 50 items divided into three subscales: eight items related to symptoms of COPD (frequency and severity), such as ” During the last year, how many severe or very bad unpleasant attacks of chest trouble have you had? “, 16 items related to an activity influenced by breathlessness such as ” how activities may be affected by your breathing “, and 26 items related to the overall impact of airway disease on psychosocial disturbances such as “I get embarrassed using my lung/respiratory medication and public”. The aggregate SGRQ is scored from 0 to 100, with higher scores indicating poorer health status. The scale has shown good internal consistency coefficients of the symptoms, activities, and impact components (Cronbach’s α = 0.94, 0.91, and 0.90, respectively) [33].

### 2.5. Data Analysis

The data were checked to identify any missing data and assessed for normality using the Shapiro–Wilk and Kolmogorov–Smirnov tests. The variables were expressed as means and standard deviations for continuous variables, numbers and percentages for categorical values. For parametric data, the independent-samples *t*-test was used for comparisons between COPD patients with no and high anxiety. The chi-squared test was used to compare categorical data. Pearson’s product-moment correlation coefficient was used to examine the relationships between the 6MWT and the main outcomes of interest. A forward multiple linear regression analysis was performed to assess the associations between the independent variables (anxiety, depression, and health-related quality of life) and the 6MWT as a dependent variable, adjusted for COPD severity. The level of statistical significance was set at *p* < 0.05. All statistical analyses were performed using Stata version 16 (Stata Corp., College Station, TX, USA).

## 3. Results

### 3.1. General Characteristics of the Participants

The patients’ physical and pulmonary function profiles are presented in Table 1. The mean patient age was 63 ± 11 years. There were no significant differences in terms of pulmonary function profile or smoking status. Based on the GOLD criteria for COPD severity, 36 (51.43%) patients had moderate COPD, with a mean percent predicated FVC_1_ of 55.98 ± 0.73.

### 3.2. Characeteristic of Psychological Factors, Functional Performance and Health-Related Quality of Life

Of the 70 patients, 46% had an AIR score of >8, and approximately 47% had a HADS-A score of >8, indicating high anxiety levels. Moreover, 49% of the patients had a HADS-D score of >8, indicating high depression levels. Data showed only 13% (*n* = 9) of the participants with depression without any anxiety, and 36% (*n* = 25) of the participants had combined anxiety and depression based on the screening tools. Furthermore, the majority of patients with depression (76.47%; *n* = 26) were in the anxiety group compared to 23.53% (*n* = 8) in the non-anxiety group. The mean aggregate HADS scores were 20.87 ± 6.13 in the high anxiety group and 9.26 ± 4.72 in the no anxiety group (*p* = 0.01). Table 2 displays the differences in depression and health status between patients with high anxiety and those with no anxiety. 

### 3.3. Effect of Anexity on Functional Performance and Health-Related Quality of Life

Patients with high anxiety levels showed significantly lower functional exercise capacity than patients with no anxiety, as reflected by the distance covered in the 6MWT (mean difference: 65.01 m; *p* = 0.01), and activities component of SGRQ (mean difference: 13.9; *p* = 0.03).. The impact of COPD was greater on patients with high anxiety level than patients with no anxiety (Table 2). Moreover, patients with high anxiety levels had poor health status compared to patients with no anxiety (56.54 ± 22.36 vs. 42.90 ± 24.76; *p* = 0.01, respectively).

### 3.4. Relationships between Functional Performance, Anxiety, Depression, and Health Status

Table 3 displays the relationships of functional performance with anxiety, depression, health status, and COPD severity. Walking performance inversely correlated with depression (*r* = −0.37), anxiety (*r* = −0.38), and health status (*r* = −0.42) and positively correlated with the FEV_1_/FVC ratio and FEV_1_ (*r* = 0.32, r = 0.45, respectively).

The multiple linear regression model with walking distance as the dependent variable adjusted for disease severity is presented in Table 4. Univariate analyses indicated that higher anxiety and depression levels and poorer health status were associated with shorter walking distances (*p* < 0.05). The final multivariate model explained 33% of the variance in functional performance as measured by the 6MWT when controlling for COPD severity (*F* = 8.97, *p* = 0.003). Anxiety, depression, and health status were significant and independent determinants of 6MWT performance.

## 4. Discussion

Poor mental health status can have a debilitating effect on functional performance. In this study, COPD patients with high anxiety and depression levels showed poorer functional performance and perceived health-related quality of life than patients with no anxiety. These findings suggest that high depression and anxiety levels and poor health status are associated with reduced functional performance among patients with COPD.

Walking is a safe and low-impact activity with substantial psychological and physiological benefits for COPD patients [34,35]. In this study, patients with high anxiety levels walked at slower speeds and covered shorter distances in the 6MWT than patients with no anxiety, although there was no difference in disease severity between the two groups. Thus, anxiety and depression are among the potential determinants of functional performance in COPD patients. Anxiety can induce hyperpnea, leading to exertional dyspnea, thus reducing exercise capacity and, ultimately, health-related quality of life [36].

The interrelationships between psychological factors and physical impairments and social behavior have been established by the International Classification of Functioning, Disability, and Health, which has been adopted by the World Health Organization disability [37]. In aligned with this context, this study’s findings show that individuals with high anxiety levels report symptoms of depression and exhibit poor functional performance. A previous cohort study suggested that the COPD stage was associated with the prevalence of anxiety ranging from 10% to 100% and depression ranging from 7% to 79% [38]. Another study reported a higher prevalence of anxiety and depression symptoms among patients with advanced-stage COPD than among patients with other advanced-stage diseases, such as cancer, AIDS, heart disease, and renal disease [39]. In contrast, in this study, there was no significant difference in COPD severity between the high anxiety and no anxiety groups.

In this study, there was a significant relationship between anxiety and depression levels and perceived poor health status. The underlying causes of these changes in mental status have yet to be elucidated and are beyond the scope of this study. Previous studies have highlighted the impacts of long-term oxygen therapy [40], poor quality of life [17], and socioeconomic status [2,41] on psychological status. Examining general anxiety and depression in mild COPD patients to identify pathophysiological factors to explain the symptoms of general anxiety. First, a hyperventilation attributed to erratic breathing patterns increases the work of breathing, which may, in turn, aggravate anxiety symptoms. Second, a cognitive behavioral model that operates by catastrophic cognitions due to the fear and misinterpretation of bodily experiences triggered by dyspnea [42].

Most previous studies have involved patients with moderate to extremely severe COPD and have found inconsistent relationships between COPD severity and mental health. This could be attributed to methodological differences in depression and anxiety measurements [3,43,44,45]. In this study, most patients showed low to moderate disease severity, although clinical depression was more severe in the high anxiety group than in the no anxiety group.

Gender is known to influence anxiety, with women being more likely to experience high levels of anxiety than men. However, we could not establish causation in this study, as most patients were men, with women accounting for only 20%. Further studies are required to assess gender-based differences in anxiety and depression levels among patients with different stages of COPD.

A previous study found that activity-related dyspnea [46], dynamic hyperinflation [47], and central hemodynamic and leg fatigue [48] affected walking performance. Another study reported that disease-related symptoms directly influenced walking performance [49]. Mental well-being could be an important factor contributing to reduced walking performance among COPD patients. In our study, patients with anxiety and depression exhibited slow walking speeds and walked short distances. Moreover, the levels of anxiety and depression negatively correlated with the distance covered in the 6MWT. Our results are in line with Di Marco et al. [50], who found that patients with depression walked short distances in the 6MWT, suggesting that depression is a potential indicator of reduced daily and maximum exercise capacity. Similarly, in a study which involved more than 1700 COPD patients [51], it has been reported that depression correlated with reduced exercise capacity. Likewise, another study found significant differences in 6MWT performance between patients with depression and individuals without depression [52].

Anxiety could be an independent factor predisposing individuals to functional limitations. Our findings confirm the impact of anxiety on poor walking performance. Similarly, Eisner et al. [3] reported that anxiety was associated with poor health outcomes, such as submaximal exercise performance. Conversely, Nguyen et al. [53] investigated the roles of depression and anxiety on physical activity in patients with COPD and found that higher anxiety levels were associated with higher levels of PA and that higher HADS-A scores correlated with higher step counts. Similarly, Biswas et al. [54] reported that anxiety and depression had no significant effects on functional status. These contradictory findings might be attributed to differences in sample characteristics, the methods used to measure study outcomes, or the severity of depression and anxiety. Therefore, further studies are needed to extensively explore the effects of psychological well-being on functional performance using objective, standardized methods.

The overall health status of COPD patients as assessed by the SGRQ, which measures the impact of the disease on overall health, daily life, and perceived well-being, could be an independent predictor of exercise intolerance. Our results show a significant relationship between health-related quality of life and walking distance, suggesting that patients who report fewer limitations walk longer distances. This relationship between health status and walking distance has also been documented in prior studies [55,56], despite the variety of methods used. Health-related quality of life and physical activity levels among COPD patients have been shown to positively correlate with better 6MWT performance and with the frequency of physical activity. These findings suggest that treatment models for COPD patients should focus not only on improving lung function but also on increasing engagement in regular activities [57]. Healthcare providers should work on not only preventing the progression of COPD but also increasing exercise capacity using various rehabilitation strategies, which must be a key goal of treatment plans for COPD patients.

Certain limitations of this study need to be acknowledged. The participants were enrolled using convenience sampling, which may have affected the generalizability of the results. Moreover, the study’s cross-sectional design precludes the establishment of causal relationships. This study investigated the potential roles of anxiety and depression as psychological predictors of impaired functional performance. However, we have no prior knowledge of the level of physical activity of these patients before their recruitment. Physical activity pattern is one of the factors that may moderate the relationship between psychological factors (anxiety and depression) and walking performance. Future studies should broaden the scope of the psychological and other health conditions that may affect the functional performance of COPD patients. Studies should also investigate the benefits of cognitive behavioral therapy, for COPD patients’ functional performance.

## 5. Conclusions

This study’s findings highlight the importance of psychological health for COPD patients. Perceived health status, anxiety, and depression are potential behavioral determinants of walking performance in this patient population. Depression and anxiety often go undetected or are undertreated in patients with COPD. Regardless of disease severity, it is essential to include psychological assessments in intervention plans and to screen patients at all stages of the disease for anxiety and depression, which significantly affect physical performance. Patients with anxiety should undergo examinations, including detailed physical and mental health histories, for further assessment and individualized treatment plans. Various validated and easy-to-use screening tools are currently available and can be integrated into the management of COPD patients by respiratory clinicians. It is imperative for healthcare providers to be aware of the interplay between mental health and physical performance and the need to educate patients about this association.

## Figures and Tables

**Table 1:** Physical characteristics of the patients.

Variable	All *n* = 70 mean ± SD	Anxiety ^1^		*p*-Value
		None *n* = 38 mean ± SD	High *n* = 32 mean ± SD	
**Age (years)**	63 ± 11	62 ± 10	65 ± 13	0.31
**Weight (kg)**	78.88 ± 15.25	76.66 ± 15.36	81.51 ± 14.93	0.81
**Height (cm)**	164.87 ± 7.90	164.03 ± 6.75	165.87 ± 9.10	0.33
**Male gender, *n* (%)**	56 (80)	32 (84)	24 (75)	0.33
				
**Pulmonary Function Profile**				
FVC (L)	2.84 ± 0.73	2.87 ± 0.66	2.81 ± 0.81	0.75
FEV_1_ (L)	1.57 ± 0.54	1.59 ± 0.51	1.55 ± 0.59	0.77
FEV_1_/FVC ratio	0.54 ± 0.11	0.54 ± 0.101	0.54 ± 0.12	0.90
FEV_1_ (% predicated)	55.98 ± 0.73	56.57 ± 12.41	55.97 ± 14.9	0.72
				
**Anxiety Inventory for Respiratory Disease**	8.17 ± 5.33	4.28 ± 2.28	12.78 ± 4.07	<0.001 *
				
**Smoking Status, *n* (%)**				
Non-smoker	14 (20)	7 (18.42)	7 (21.88)	0.88
Smoker	17 (24.29)	10 (26.32)	7 (21.88)	
Ex-smoker	39 (55.71)	21 (55.26)	18 (56.25)	
Male gender, *n* (%)	56 (80)	32 (84.21)	24 (75)	0.33
				
**GOLD stage, *n* (%)**				
I	4 (5.71)	2 (5.26)	2 (6.25)	0.64
II	36 (51.43)	22 (57.89)	14 (43.75)	
III	27 (38.57)	13 (34.21)	14 (43.75)	
IV	3 (4.29)	1 (2.63)	2 (6.25)	

^1^ Independent-samples *t*-test; * statistically significant (*p* < 0.05). **Abbreviations:** GOLD: Global Initiative for Chronic Obstructive Lung Disease.

**Table 2:** Comparison of functional performance and psychological outcomes between patients with no anxiety and patients with high anxiety levels (*n* = 70).

Variable	Anxiety ^1^		*p*-Value
	None (*n* = 38) Mean ± SD	High (*n* = 32) Mean ± SD	
**St. George’s Respiratory Questionnaire**			
Symptoms component	49.96 ± 22.81	57.81 ± 23.87	0.16
Activities component	49.65 ± 28	63.55 ± 25.45	0.03 *
Impact component	36.97 ± 27.26	52.06 ± 25.08	0.01 *
Total	42.90 ± 24.76	56.54 ± 22.36	0.01 *
			
**Hospital Anxiety and Depression Scale**			
Anxiety items	4.31 ± 2.78	10.96 ± 4.01	0.001 *
Depression items	4.94 ± 2.95	9.90 ± 3.41	0.001 *
6MWT distance (m)	373.76 ± 106.56	308.75 ± 120.16	0.01 *

^1^ Independent-samples *t*-test; * statistically significant (*p* < 0.05). **Abbreviations:** 6MWT: six-minute walk test.; m: meters; SD, standard deviation.

**Table 3:** A Matrix of the Pearson correlation between the Walking Performance, and personal psychological characteristics among the study respondents.

Variables	Correlation Coefficients					
	1	2	3	4	5	6
6 MWT distance	1					
AIR scale	−0.38 *	1				
HADS-d	−0.37 *	0.85 *	1			
SGRQ	−0.42 *	0.42 *	0.43 *	1		
FEV_1_(Liters)	0.45 *	−0.19	−0.173	−0.45 *	1	
FEV_1_/FVC ratio	0.32 *	−0.20	−0.20	−0.23 *	0.68 *	1

* Correlation is significant at *p* value of 0.01. **Abbreviation:** AIR scale, Anxiety Inventory for Respiratory Disease; HADS, Hospital anxiety and depression scale assessment; SGRQ, St George’s Respiratory Questionnaire.

**Table 4:** Multiple linear regression analysis for the 6 MWT distance as dependent variable.

Model	Predictors ^a^	Coefficients						R^2^
		β	B	T	*p* Value	95% CI		
						Lower	Upper	
Model *	(Constant)	-	386.28	4.67	<0.001 *	221.13	551.43	0.33
	HADS	−0.27	−3.99	−2.54	0.01 *	−7.13	−0.85	
	AIR	−0.27	−5.97	−5.97	0.01 *	−10.65	−1.29	
	SGRQ	−0.28	−1.35	−2.55	0.01 *	−2.41	−0.29	
	**F**							
		8.97						
	***p*-value**	0.003 *						

**Abbreviations**: B: unstandardized beta “regression coefficient”; β: standardized beta. a. Predictors: AIR scale, Anxiety Inventory for Respiratory Disease; HADS, Hospital anxiety and depression scale assessment; SGRQ, St. George’s Respiratory Questionnaire. * Regression model adjusted for the COPD severity and significant level set at *p* < 0.05.

## Data Availability

The identified datasets analyzed during the current study are available from the corresponding author on reasonable request.
